# Biodegradation of Nonylphenol Monopropoxyethoxylates

**DOI:** 10.1007/s11743-014-1652-8

**Published:** 2014-12-04

**Authors:** Agnieszka Zgoła-Grześkowiak, Tomasz Grześkowiak, Andrzej Szymański

**Affiliations:** Institute of Chemistry and Technical Electrochemistry, Poznan University of Technology, Berdychowo 4, 60-965 Poznan, Poland

**Keywords:** Nonylphenol monopropoxyethoxylates, Biodegradation

## Abstract

Aerobic biodegradation behavior of nonylphenol monopropoxyethoxylates was investigated in two tests with different inocula-sewage sludge and river water. Both primary biodegradation and formation of different biodegradation intermediates were studied. Primary biodegradation of nonylphenol monopropoxyethoxylates was relatively fast and complete with the sewage sludge as the inoculum. On the other hand, biodegradation with river water as the inoculum was slower and primary biodegradation in this test reached only about 60 % during almost 50 days. The biodegradation intermediates from both oxidative and non-oxidative pathways were found. In the non-oxidative route monopropoxy poly(ethylene glycol)s were observed which indicate existence of the central fission biodegradation pathway. In the oxidative pathway carboxylic acids were identified. The biodegradation intermediates identified with the use of high performance liquid chromatography with mass spectrometric detection persisted for many days in both tests.

## Introduction

Non-ionic surfactants belong to the most important chemicals that are used in industrial, agricultural, commercial and household applications. One of the major group of non-ionic surfactants are alkylphenol ethoxylates (APEO) which are commonly applied for cleaning, lubrication, defoaming, emulsifying, wetting and de-wetting [1]. They are used in various cleaning products, paints, pesticide formulations, textile processing, paper manufacturing, metal processing and many other applications [1, 2]. On the other hand, use of APEO is restricted in some countries due to their incomplete biodegradation under normal environmental conditions resulting in formation of more persistent and problematic metabolites. The European Union legislated limitations for the use of nonylphenol ethoxylates (NPEO) and nonylphenol (NP) [3]. Regulations concerning environmental levels of these surfactants were also issued by the Canadian Council of Ministers of the Environment [4–6]. US Environmental Protection Agency regulates environmental level of nonylphenol which is the intermediate product of NPEO biodegradation [7] and European Union issued regulations for allowable concentration in surface waters of both nonylphenol and octylphenol [8]. However, APEO are still used in many countries by virtue of their excellent performance and low production costs. Among APEO, NPEO are the most commonly used isomers, make up circa 80–85 % APEO produced [9]. The annual worldwide usage of APEO in the year 2000 was about 700 kilotons [10]. Recent data on usage of APEO are not available, both at the global and at the European Union level. Precise information dates back before the restrictions introduced by the European Union. Current emission of NPEO to the environment in the European Union is evaluated at above 11 kilotons per year [11]. Recent annual North American consumption is estimated to 160 kilotons [12].

APEO are not classified as highly toxic substances but rather their estrogenic potential is taken into consideration. Initial biodegradation of APEO occurs rather rapidly with loss of surfactant properties. It was shown in many papers that their breakdown products are ubiquitous in the environment [13–15] and are more toxic than the parent compounds [16]. The intermediate products of APEO biodegradation including alkylphenols, their short-chained ethoxylates and short-chained carboxylated metabolites are known from their estrogenic properties [17–19] and ability to bioaccumulate in aquatic organisms [20–22]. Therefore, many studies have been undertaken to assess biodegradation of APEO in different tests [23–28] and in real sewage treatment plants [29]. The environmental impact of the intermediate products of their biodegradation [17] and contamination of rivers [14, 15, 30–33], lakes [34–36] and even drains [37] by these compounds were extensively studied.

Nevertheless, despite many environmental problems connected with APEO, their usefulness makes them attractive in many industrial applications. Given the great demand for APEO a new kind of nonionic surfactant was recently introduced which is capable of replacing APEO. The nonylphenol monopropoxyethoxylates (NPPOEO) can be used in applications similar to NPEO [38]. It is considered, that introduction of one propoxy group to the molecule reduced their environmental impact, because their short-chained intermediate products of biodegradation are indicated as having no estrogenic character [38]. This makes the NPPOEO very attractive for industry, even though formation of estrogenic nonylphenol during biodegradation of NPPOEO cannot be excluded.

In this paper the biodegradation of a new substance belonging to the NPPOEO group was tested under aerobic conditions. Both primary biodegradation and formation of intermediate products of biodegradation were studied with the use of HPLC with mass spectrometric detection. The results obtained will give an interesting insight in biodegradation of these new surfactants.

## Materials and Methods

### Reagents and Chemicals

The new nonionic surfactant belonging to the NPPOEO group was obtained from Oil Chem Technologies (Sugar Land, TX, USA) under the commercial abbreviated name NP-PC-20EO. The tested surfactant contains one propoxylene subunit and an ethoxylene chain with an average ethoxylation degree of 20. Nonylphenol was obtained from Sigma-Aldrich (St. Louis, MO, USA). MS grade and HPLC gradient grade acetonitrile was from Sigma-Aldrich. Water was prepared by reverse osmosis in a Demiwa system from Watek (Ledec nad Sazavou, The Czech Republic), followed by double distillation from a quartz apparatus from Heraeus (Germany). Only freshly distilled water was used. All the chemicals used for preparing media in both tests were from POCH (Gliwice, Poland).

### Biodegradation Study (Modified OECD Screening Test)

Static screening tests for ready biodegradability in aerobic conditions were performed according to the OECD method 301E (Modified OECD Screening Test) [39]. NPPOEO with one propoxylene subunit and an average ethoxylation degree 20 was tested. Surfactant concentration of 10 mg L^−1^ was applied in both tests. The medium used in both tests consisted of mineral components (KH_2_PO_4_, K_2_HPO_4_, Na_2_HPO_4_·2H_2_O, NH_4_Cl, CaCl_2_, MgSO_4_·7H_2_O, FeCl_3_·6H_2_O, MnSO_4_·4H_2_O, H_3_BO_3_, ZnSO_4_·7H_2_O (NH_4_)_6_Mo_7_O_24_), and yeast in appropriate concentrations [39]. The inoculum for the first test was taken from a small rural sewage treatment plant (STP) located in Tarnowo Podgórne which treats 3,000 m^3^ day^−1^ of sewage. The inoculum for the second test was taken from the middle of the mainstream of the River Warta in Poznań near the St. Roch Bridge. The tests were performed in 2-L bottles and lasted for 49 days. A test for possible abiotic degradation in sterile controls containing no inoculum was not required due to excellent chemical stability of alkylphenol alkoxylates. The samples from both tests were subjected to HPLC–MS analysis and the biodegradation rate was determined.

### HPLC–MS Analysis of Tested Surfactant and Ethoxylated Intermediates of Its Biodegradation

A chromatographic system UltiMate 3000 RSLC from Dionex (Sunnyvale, CA, USA) was used. The 5-µL samples were injected into a CN column (100 mm × 3 mm I.D.; 1.8 µm) from Agilent Technologies (Santa Clara, CA, USA). The mobile phase employed in the analysis consisted of 5 × 10^−3^ mol L^−1^ ammonium acetate in water and acetonitrile at a flow rate of 0.3 mL min^−1^ at 35 °C. Gradient elution was performed by linearly increasing the percentage of organic modifier from 60 to 70 % in 4 min and then to 95 % in 1 min and it was maintained at 95 % for 3 min. A pre-run time of 4 min was done before the next injection. The chromatographic system was connected to the API 4000 QTRAP triple quadrupole mass spectrometer from AB Sciex (Foster City, CA, USA). The LC column effluent was directed to the electrospray ionization source (Turbo Ion Spray). The Turbo Ion Spray source operated in positive ion mode. The following settings for the ion source and mass spectrometer were used: curtain gas 20 psi, nebulizer gas 40 psi, auxiliary gas 40 psi, temperature 450 °C, ion spray voltage 4,500 V, declustering potential 50 V. The mass spectra were recorded using mass scan in 100–1,800 *m*/*z* range. The results were calculated semi-quantitatively because analytical standards of particular NPPOEO homologues and their biodegradation intermediates are not available.

### HPLC–MS Analysis of Nonylphenol

The analysis was performed by the previously described procedure [40]. Briefly, a chromatographic system UltiMate 3000 RSLC was used. The 10-µL samples were injected into a phenyl-hexyl column (50 mm × 3 mm I.D.; 1.8 µm) from Agilent Technologies. The mobile phase employed in the analysis consisted of 5 × 10^−3^ mol L^−1^ ammonium acetate in water (A) and acetonitrile (B) at a flow rate of 0.3 mL min^−1^ at 35 °C. For the analysis of NP the following gradient was used: 0 min. 60 % B; 5 min. 70 % B; 6 min. 95 % B; 8 min. 95 % B. A pre-run time of 4 min was done before the next injection. The chromatographic system was connected to the API 4000 QTRAP mass spectrometer. The Turbo Ion Spray source operated in negative ion mode. The following settings for the ion source and mass spectrometer were used: curtain gas 20 psi, nebulizer gas 40 psi, auxiliary gas 40 psi, temperature 480 °C, ion spray voltage −4,500 V, declustering potential −80 V and collision gas set to medium. The dwell time for each mass transition detected in the selected reaction monitoring mode was set to 100 ms. The quantitative transition was from 219.3 to 133.3 *m*/*z* at collision energy set to −48 V and the confirmatory transition was from 219.3 to 147.3 *m*/*z* at collision energy set to −35 V. The linearity of the method was tested in the range 1–1,000 µg L^−1^ and the coefficient of determination was 1.00. Accuracy was 100 % because only filtration of samples was done before their HPLC–MS analysis, and precision was acceptable with relative standard deviation not higher than 10 %.

## Results and Discussion

**Fig. 1:**
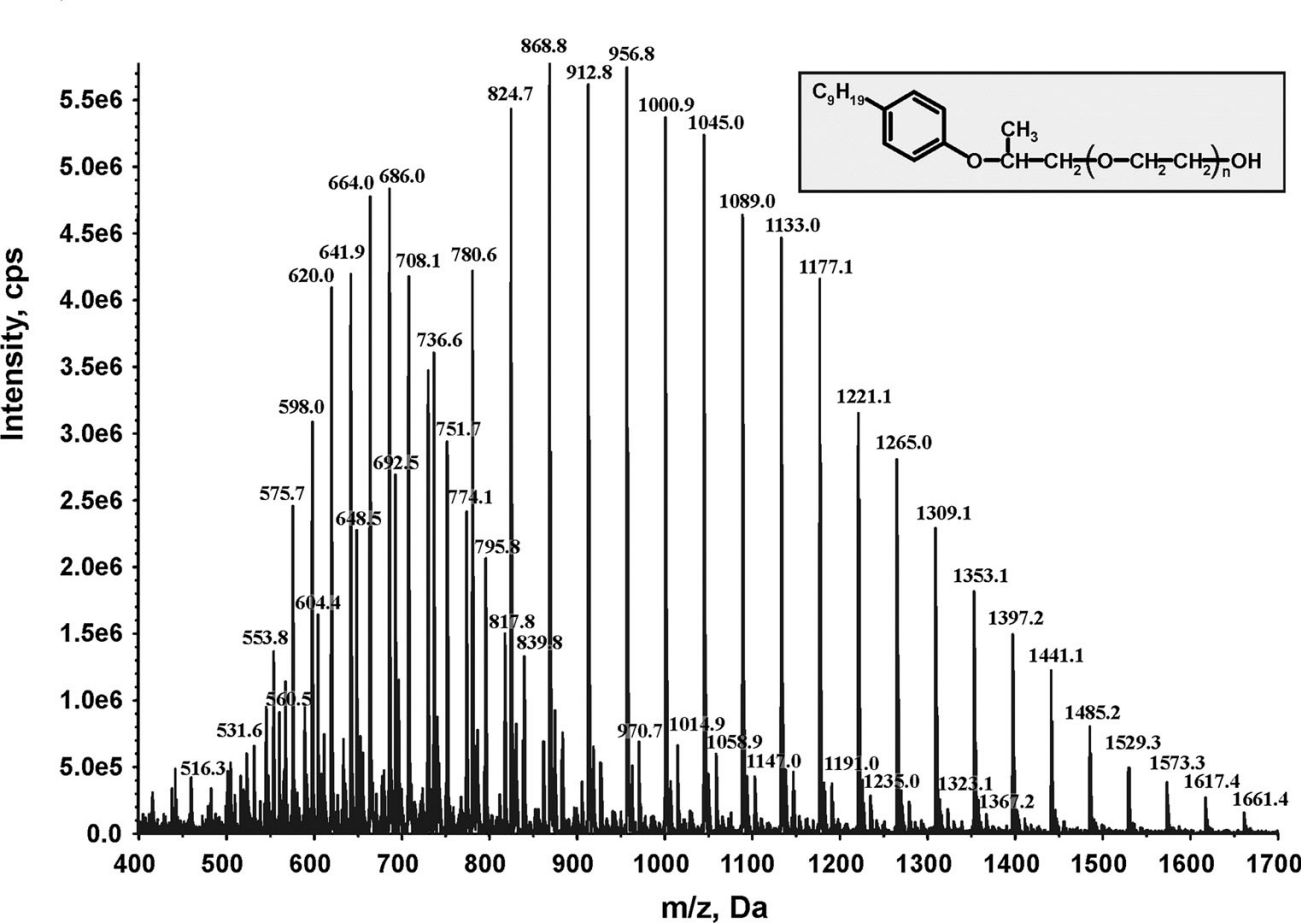
Mass spectrum of the tested nonylphenol monopropoxyethoxylate

Before performing the biodegradation tests, the chemical composition of the tested NPPOEO was confirmed by mass spectrometry measurements. Figure 1 shows the simplified structural formula of NP-PC-20EO and its mass spectrum with typical distribution of signals for homologues of NPPOEO with different number of ethoxylene subunits in the molecule.

**Fig. 2:**
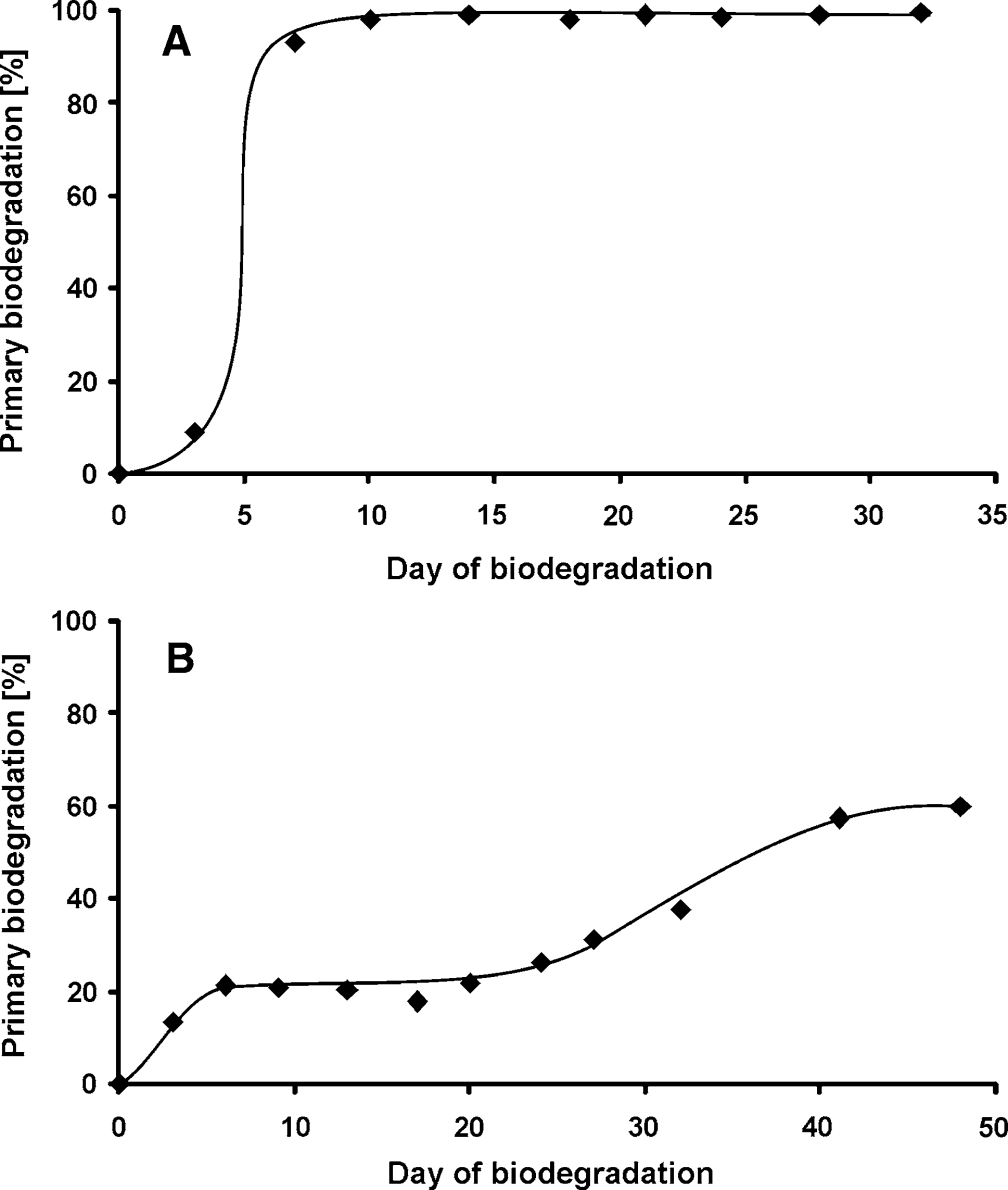
Primary biodegradation degree of nonylphenol monopropoxyethoxylate during the biodegradation test **a** inoculum from sewage treatment plant, **b** inoculum from river water

The biodegradation of the tested surfactant differs considerably in the two tests performed in this study, depending on the applied inoculum. The primary biodegradation which leads to loss of surface active properties was completed in 9 days in the first test (with activated sludge taken from the STP as inoculum). In contrast to the results of the first test, in the second test (with river water as the inoculum) the primary biodegradation reached maximally about 60 % in nearly 50 days, whereas after 9 days was only about 20 % (Fig. 2).

These differences may result from the larger biodiversity and number of bacteria in activated sludge (the first test) compared to the river water (the second test). This can be considered in two respects. On the one hand, the qualitative factor is important, because a much greater number of different bacterial strains can be found in the activated sludge than in the river water. Hence, there is a greater likelihood that there will be more strains capable of NPPOEO biodegradation in the first test than in the second one. On the other hand, the quantitative factor plays an important role, because the growth of bacterial colonies capable of NPPOEO biodegradation deepens the observed differences in the degree of NPPOEO biodegradation in activated sludge and river water.

**Fig. 3:**
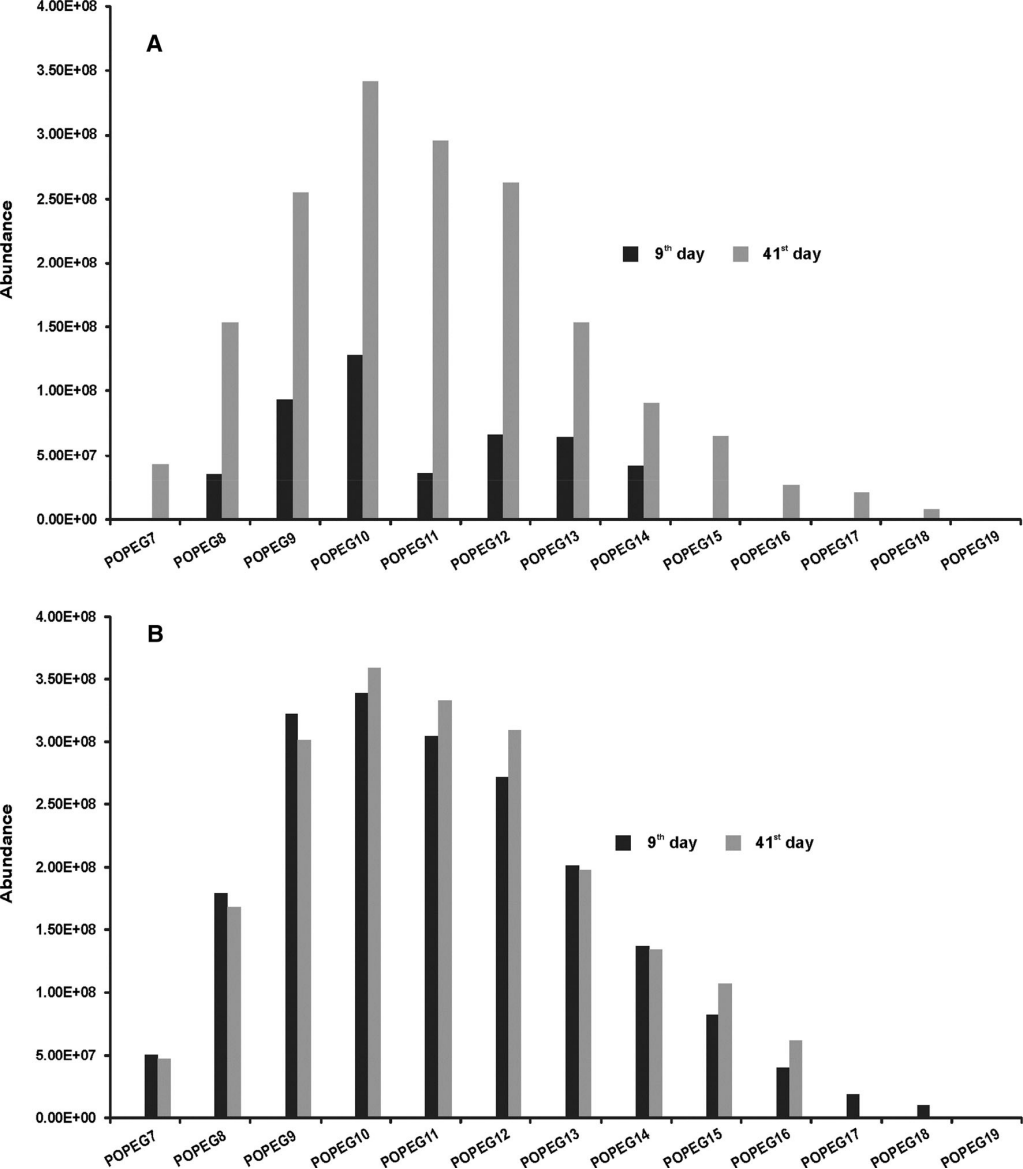
Monopropoxy poly(ethylene glycol)s found during the biodegradation test of nonylphenol monopropoxyethoxylate. **a** Inoculum from a sewage treatment plant, **b** inoculum from river water

A relatively high amount of monopropoxy poly(ethylene glycol)s (POPEG) was observed in biodegradation products from both tests already in the first days after their initiation (Fig. 3). The amount of POPEG formed in the beginning of the first test was considerably lower than in the second one. However, no difference was found in the final days of the tests. There was also no chain shortening of POPEG over the duration of the tests. The presence of POPEG among the biodegradation products indicates the existence of the biodegradation pathway by central fission on the phenolic oxygen atom.

**Fig. 4:**
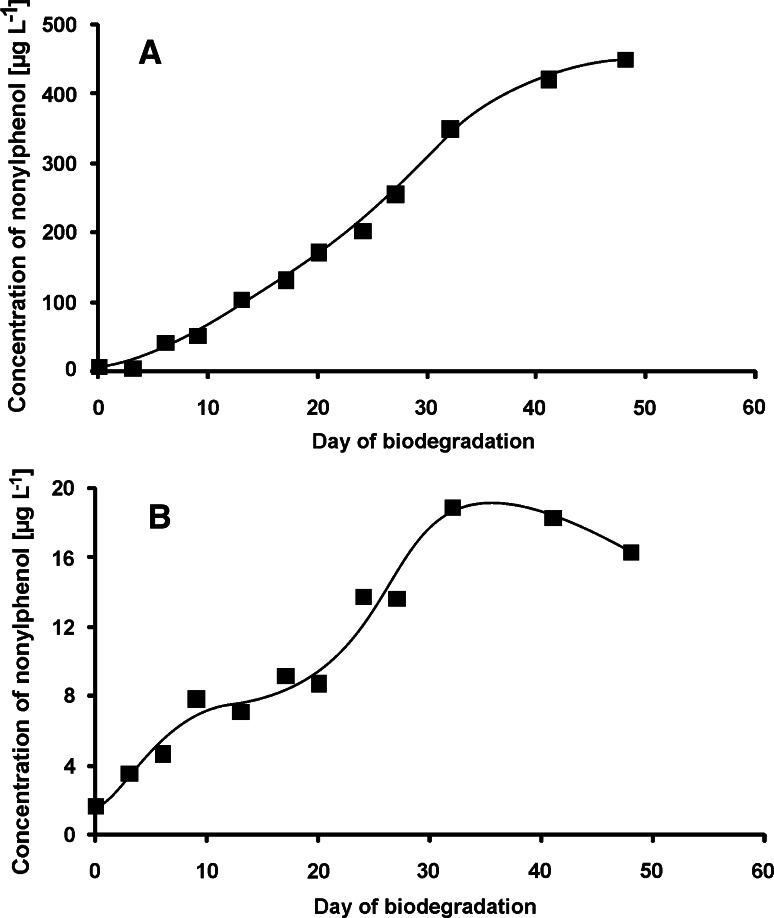
Concentration of nonylphenol during the biodegradation test of nonylphenol monopropoxyethoxylate. **a** Inoculum from a sewage treatment plant, **b** inoculum from river water

As a result of the central fission mechanism, formation of nonylphenol in both tests is inevitable. Indeed, the presence of nonylphenol was confirmed in both tests (Fig. 4), what also confirms the correctness of the assumption that NPPOEO biodegradation occurs through the central fission. However, amount of nonylphenol formed in the first test was considerably higher than in the second one and reached about 450 µg L^−1^ at the end of the test. On the contrary, in the second test the highest concentration of nonylphenol was noted on the 31st day. On the following days, the amount of nonylphenol in the biodegradation liquor decreased even though the primary biodegradation of NPPOEO increased in this period of time. A gradual increase of nonylphenol in the first test may mean that the processes of its formation are kinetically faster than the processes of its biodegradation. Another reason could be a long adaptation time of activated sludge bacterial strains to biodegradation of nonylphenol. Theoretically, observed effect may also result from the fact, that the bacteria from the STP are not able to transform nonylphenol at all, but in practice it is unlikely due to high biodiversity in the activated sludge. On the contrary, bacteria from the river water used in the second test biodegrade nonylphenol formed during the experiment. Its amount decreases after 30 days of the test which can be attributed to high growth of bacterial strains degrading nonylphenol.

**Fig. 5:**
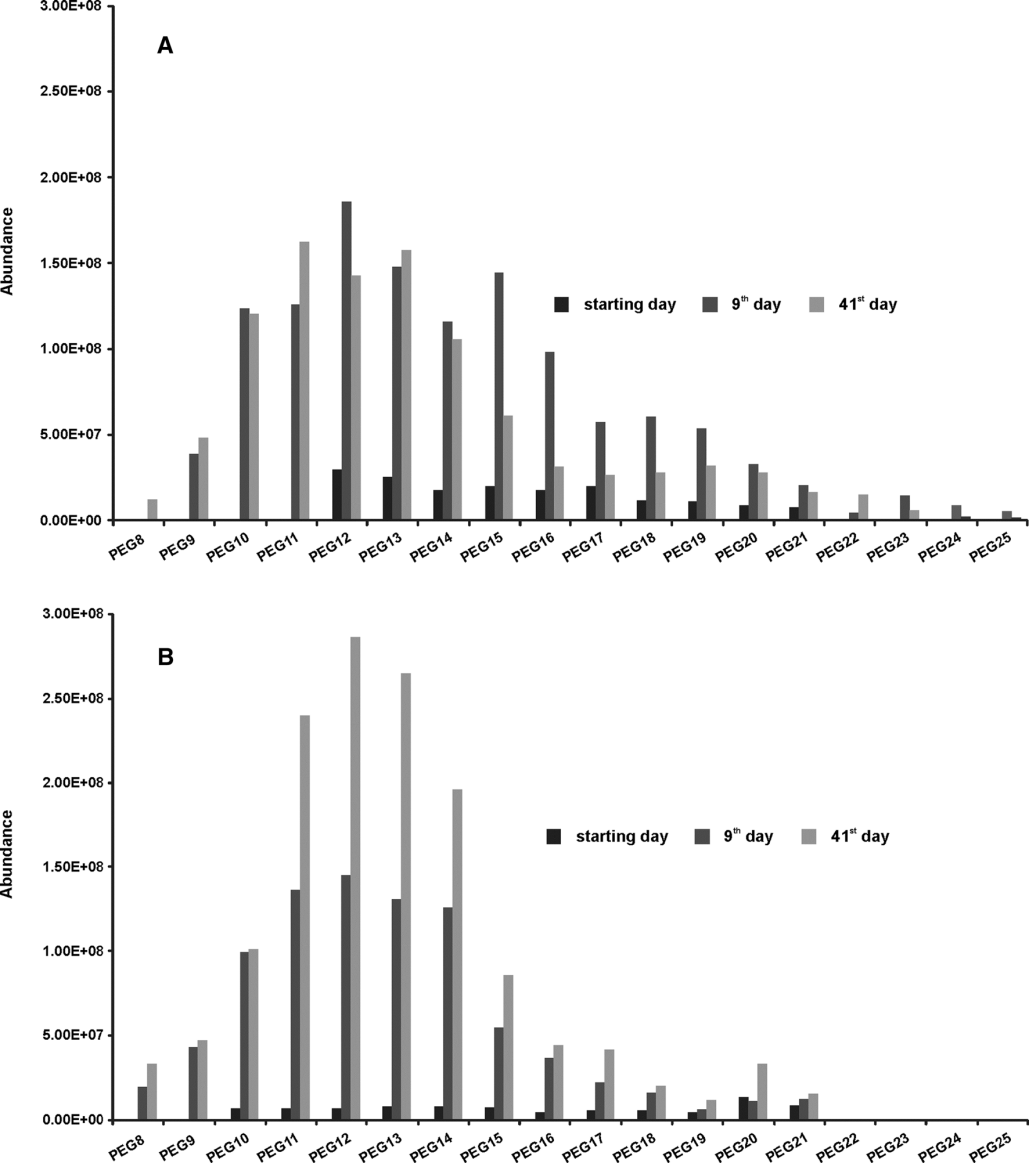
Poly(ethylene glycol)s found during the biodegradation test of nonylphenol monopropoxyethoxylate. **a** Inoculum from sewage treatment plant, **b** inoculum from river water

Formation of poly(ethylene glycol)s (PEG), and their accumulation were evident in both tests (Fig. 5). What is important, the propoxynonylphenol was not found in the presence of PEG, indicating that the PEG are not formed directly from the molecules of NPPOEO, but as a results of further POPEG biodegradation. Definitely a greater tendency to accumulate in the biodegradation liquor show PEG formed in the second test. The abundance of MS signals for individual homologues of PEG in the last day of this test was the highest (Fig. 5), in comparison to the abundance of corresponding signals in the initial, and 9th day of the test. In the first biodegradation test substantial accumulation of PEG occurred only in the early days of the test, as indicated by higher abundance of MS signals in the mass spectrum after 9th day of the test (Fig. 5), in comparison to the abundance of corresponding MS signals in the mass spectrum in the initial day of the test. Starting from about the 9th day of the first test, similar amount of PEG was found on subsequent days until the end of the test. It is worth to stress, that tendency to accumulate PEG during the tests indicate their poor susceptibility to further biodegradation under the specified conditions of biodegradation tests.

Moreover, particularly in the mass spectrum for the PEG fractions after the 9th and final day of the first test, yet another effect is shown. The signals of higher PEG homologues (containing not less than 15 ethoxylene subunits in the molecule) have a significantly higher MS abundance in the mass spectrum obtained on the 9th day of the test, than on the last day (Fig. 5). Considering that the primary biodegradation was completed in 9 days in the first test, and that the abundance of homologues with low ethoxylation degree (below 15) are virtually identical in the mass spectra of the 9th and final day of the test, it can be concluded that the observed effect of significant reduction of the abundance of homologues with a high ethoxylation degree (above 15), is a consequence of domination of the ethoxylene chain shortening mechanism in PEG biodegradation process. Because the biodiversity of bacteria and a number of bacterial strains capable to biodegradation of NPPOEO was smaller in river water (the second test) in comparison to the activated sludge (the first test), the primary biodegradation rate in the second test was substantially lower, and biodegradation process was not completed even in the final day of the test. In this situation, the biodegradation products were being enriched by PEG in a continuous manner in each day of the second test, and consequently the abundance of PEG homologue signals obtained in the last days of this test was much higher than in the 9th day (Fig. 5). As a result, in the second biodegradation test the ethoxylene chain shortening of PEG, has not been documented as convincingly as in the first one.

**Fig. 6:**
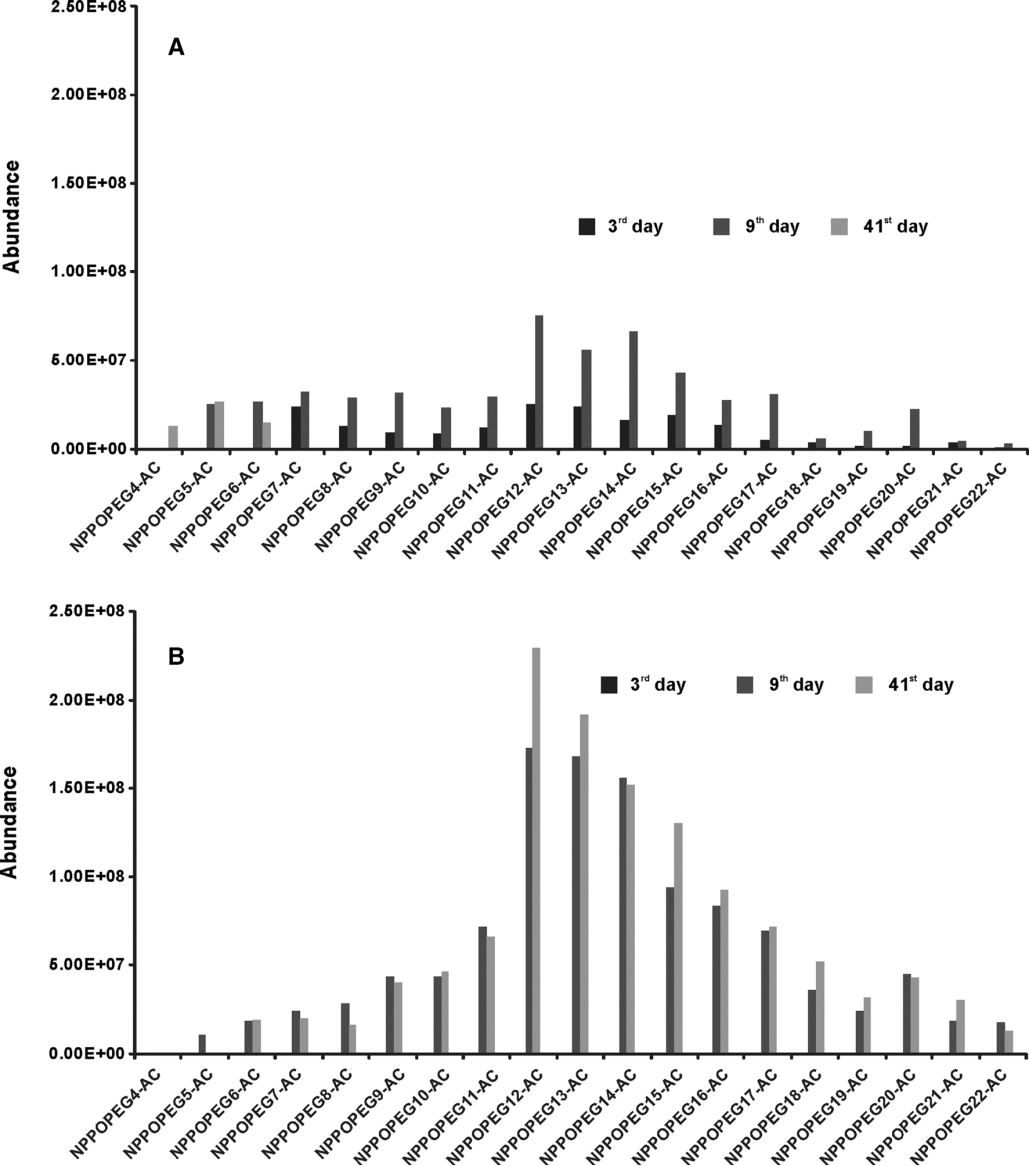
Carboxylic intermediate products of nonylphenol monopropoxyethoxylates biodegradation found during the biodegradation test of nonylphenol monopropoxyethoxylate. **a** Inoculum from a sewage treatment plant, **b** inoculum from river water

**Fig. 7:**
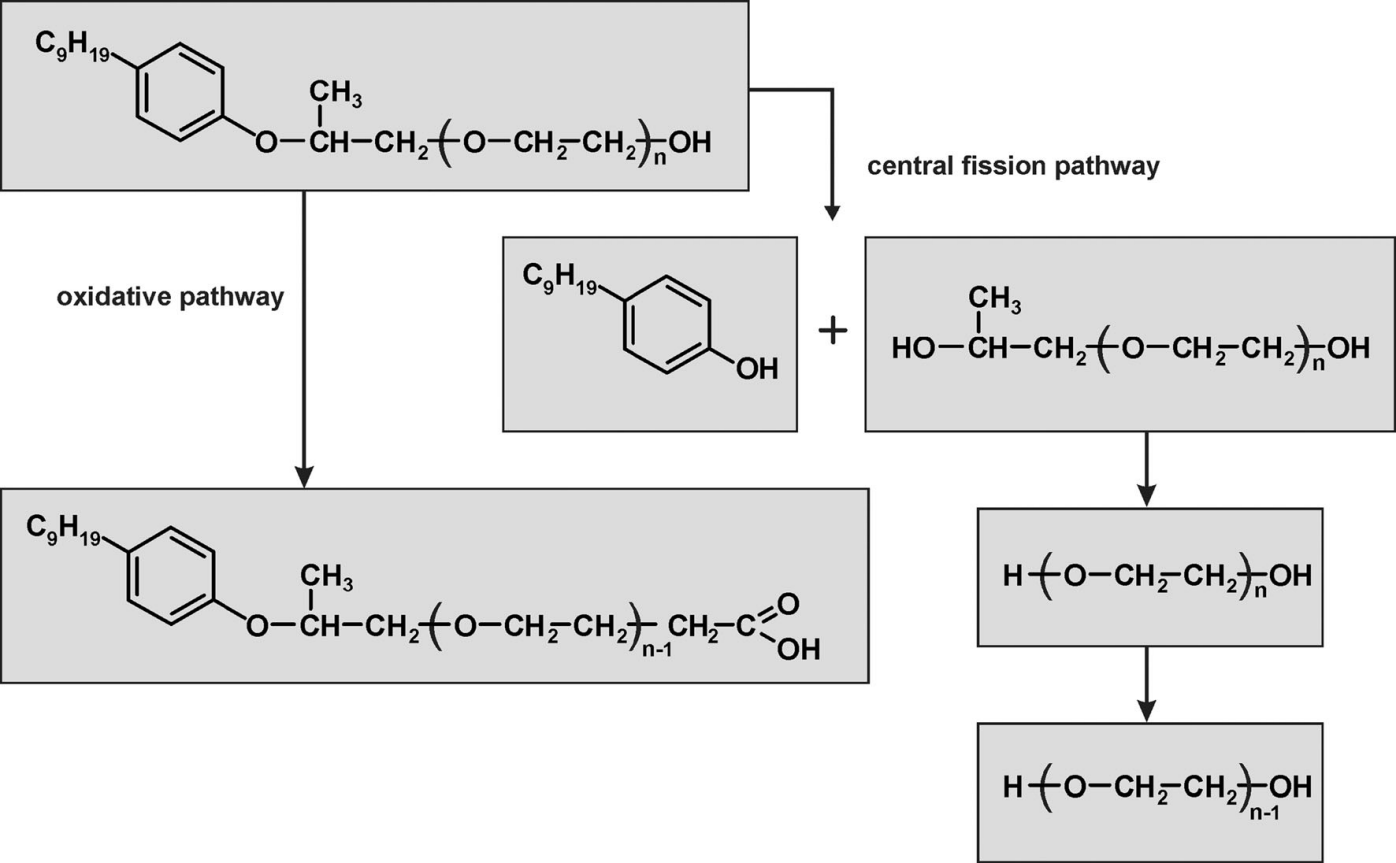
The postulated biodegradation mechanism of nonylphenol monopropoxyethoxylates

The central fission process is not the only one biodegradation pathway for NPPOEO. The acidic (carboxylic) intermediate products of NPPOEO biodegradation (NPPOEO-AC) which are generated by the oxidation of the final hydroxyl group of NPPOEO ethoxylene chain to the carboxyl group were also found in both tests (Fig. 6). In the first biodegradation test NPPOEO-AC were found already in the 3rd day. A higher amount of these compounds was noted in a few next days, but only small amount was found in the last days of this test. Substantial shortening of NPPOEO-AC ethoxylene chain was also noted. In the second biodegradation test NPPOEO-AC were not found in such early stage, as it was in the first one. However, they were found in later days. It is noteworthy to underline that beginning from the 6th day of the test abundance of these compounds in the second test was much higher than in the first one. A similar amount of carboxylic compounds was found from the 6th day till the end of the second test and no ethoxylene chain shortening was observed. On the basis of these results and considering that POPEG were found in both tests it can be assumed that the central fission was the main biodegradation mechanism in the first test while in the second test both mechanisms were playing substantial role. Although, depending upon the applied inoculum in the two performed tests, two different biodegradation pathways were observed with varying intensity, one general biodegradation scheme can be proposed (Fig. 7). This scheme is similar to those proposed in the literature for NPEO [23]. It takes into account the possibility of NPPOEO biodegradation both by the oxidative shortening of ethoxylene chain, what for many years is considered as typical for nonylphenol ethoxylates, and by central fission of their molecules, what is a new trend in the interpretation of nonylphenol ethoxylates biodegradation processes [23].

## Conclusion

A different biodegradation rate of NPPOEO was observed in tests with inoculum from STP and river water. However, the biodegradation products formed in these tests were similar. POPEG found in the biodegradation liquor in both tests prove existence of the central fission mechanism for the NPPOEO biodegradation that was previously postulated for the NPEO. This mechanism is not the only one, because the acidic intermediate products of NPPOEO biodegradation were also found. The amounts of nonylphenol formed in the two tests were different. A concentration of nonylphenol about twenty times higher was noted in the test with sewage sludge as inoculum than in the test with river water as inoculum.
